# Light-Dependent Regulatory Interactions between the Redox System and miRNAs and Their Biochemical and Physiological Effects in Plants

**DOI:** 10.3390/ijms24098323

**Published:** 2023-05-05

**Authors:** Zsolt Gulyás, András Székely, Kitti Kulman, Gábor Kocsy

**Affiliations:** 1 Agricultural Institute, Centre for Agricultural Research ELKH, Department of Biological Resources, 2462 Martonvásár, Hungary; 2 Max Planck Institute of Molecular Plant Physiology, Am Mühlenberg 1, 14476 Potsdam, Germany

**Keywords:** antioxidants, biogenesis of miRNAs, development, growth, light intensity, light spectrum, reactive oxygen species, stress response

## Abstract

Light intensity and spectrum play a major role in the regulation of the growth, development, and stress response of plants. Changes in the light conditions affect the formation of reactive oxygen species, the activity of the antioxidants, and, consequently, the redox environment in the plant tissues. Many metabolic processes, thus the biogenesis and function of miRNAs, are redox-responsive. The miRNAs, in turn, can modulate various components of the redox system, and this process is also associated with the alteration in the intensity and spectrum of the light. In this review, we would like to summarise the possible regulatory mechanisms by which the alterations in the light conditions can influence miRNAs in a redox-dependent manner. Daily and seasonal fluctuations in the intensity and spectral composition of the light can affect the expression of miRNAs, which can fine-tune the various physiological and biochemical processes due to their effect on their target genes. The interactions between the redox system and miRNAs may be modulated by light conditions, and the proposed function of this regulatory network and its effect on the various biochemical and physiological processes will be introduced in plants.

## 1. Introduction

Light is necessary for optimal plant growth and development and it is the most important energy source for biomass production [1]. Plants utilize the blue and red wavelengths of the light spectrum for photosynthesis. They can respond to the wavelengths from the ultraviolet (UV, 280–400) to the far-red (FR, 700–800) regimen of the spectrum. Light is perceived by different types of photoreceptors, such as phytochromes (PHYs), cryptochromes (CRYs), phototropins (PHOTs), and UV RESISTANCE LOCUS 8 (UVR8) [2,3]. PHYs absorb red and far-red light; CRYs perceive green and blue light signals. PHOTs also take in blue light, and the UVR8 perceives UV-B radiation. CRYs and PHYs play important roles in the regulation of plant light responses, for example, light-dependent seed germination, de-etiolation, shade avoidance, stomatal development, circadian rhythm, and photoperiodic flowering [4]. Besides these functions, light conditions significantly regulate the defence responses of plants, particularly the induction of locally and systemic acquired resistance and the detoxification mechanisms [5].

Spatial (latitude, altitude) and temporal (daily, seasonal) changes in light intensity and spectrum affect the formation of reactive oxygen species (ROS) and the activity of the antioxidant system [6,7]. This system maintains redox homeostasis by the removal of the excess ROS [8,9]. ROS are produced continuously by plant aerobic metabolism in different cell compartments, including chloroplastids, mitochondria, and peroxisomes [10]. They are two-faced molecules in plants since they can cause DNA damage and cell death; on the other hand, they are significant secondary messenger molecules in signalling pathways [11,12,13]. The main ROS are hydrogen peroxide (H_2_O_2_), superoxide radical (O_2_^.−^), hydroxyl radical (HO^.^), and singlet oxygen (^1^O_2_). The most important antioxidants are ascorbic acid (AsA), glutathione (GSH), tocopherol, carotenoids, flavonoids, thioredoxins (TRXs), peroxiredoxins (PRXs), catalase (CAT), superoxide dismutase (SOD), ascorbate peroxidase (APX), glutathione reductase (GR) and glutathione S-transferases (GSTs) [6,14,15]. Antioxidants, together with ROS, participate in the light intensity- and spectrum-dependent modulation of metabolism, which affects growth, development, and stress response [6].

Important regulators of the redox system are the microRNAs (miRNAs) [16]. They are 20–24 nucleotides long, non-coding single-stranded small RNAs [17]. Regarding their function, miRNAs regulate gene expression via transcriptional gene silencing (TGS), preferably through the post-transcriptional gene silencing (PTGS) pathway. TGS takes place via DNA methylation, causing histone modification [18]. PTGS occurs in two different ways. miRNAs can inhibit the translation when the miRNA–protein complex binds to its target transcript, or cleave the target mRNA sequences [19]. The biogenesis of miRNAs begins with their transcription by RNA polymerase II (Pol II) from the miRNA genes [20]. The product is the primary miRNA (pri-miRNA) containing a 5′ cap and a 3′ poly (A) tail. It is processed by the core microprocessor complex consisting of a type III RNAse, DICER-LIKE1 (DCL1), a zinc finger protein SERRATE (SE), and a dsRNA binding protein HYPONASTIC LEAVES1 (HYL1) to a hairpin-shaped precursor-miRNA (pre-miRNA) that lacks 5′ and 3′ ends. During further processing, the DCL1 truncates the loop from the miRNA, creating a miRNA/miRNA* duplex which will be methylated on the 3′ ends by the HUA ENHANCER1 (HEN1) in order to prevent its degradation. After the degradation of miRNA*, the single-stranded miRNA will be loaded on the ARGONAUTE1 (AGO1), and a miRNA-Induced Silencing Complex (miRISC) will be formed. It will be guided by the miRNA to its target mRNA resulting in the regulation of the gene expression [17]. It was found that spatial and temporal regulation of the miRNAs might be necessary to maintain the appropriate homeostasis in the different organisms [21,22]. Furthermore, it was shown that miRNAs are able to move long-distance in plants, coordinating distal tissues [23,24]. It was reported that miRNAs can not only downregulate the expression of their target genes, but can also upregulate them, as observed in mammals and plants [25,26,27,28].

## 2. Modulation of the Redox System by Light

### 2.1. Effect of Light Intensity on the Redox System

High light intensity induces photo-oxidative stress and the greater formation of ROS in chloroplasts [6,29], which occurs under natural conditions in high mountains with increasing altitude because of the simultaneous increase of light intensity [30]. It means such intensity, which is greater than the optimal one for photosynthesis [31]. With increasing light intensity, the photosynthetic reaction centers became saturated with energy, leading to the reduction of energy fraction used in photosynthesis and the subsequent accumulation of excess energy. If the non-photochemical quenching (that has a linear relationship with the tolerated light intensity and prevents the excitation of chlorophyll) is not enough to remove excess energy in the case of extreme (super) high light intensity, ^1^O_2_ will be produced in excess. It is formed from the ground-state molecular oxygen (^3^O_2_) because of its interaction with excited (triplet) state chlorophylls within photosystem II (PSII) in thylakoid membranes [32,33]. In addition, the reduction of ^3^O_2_ by PSI leads to the generation of O_2_^.−^, from which H_2_O_2_ will be synthesized on the thylakoid membranes (stromal side) in a spontaneous reaction or by SODs. ROS produced in chloroplasts greatly influence the excess light-induced leaf senescence, which is accompanied by the transcription of senescence-associated genes [32]. Among the first responses to excess light (2-day 1500 µmol m^−2^ s^−1^ PFD, control 150 PFD), the ^1^O_2_-dependent activation of gene expression was observed within 24 h in Arabidopsis, when the marker genes for H_2_O_2_ were not activated yet [34]. H_2_O_2_ may be involved in the control of senescence during long exposure to excess light. The intensity of light, having a damaging effect, differs between plant species and depends on the environmental conditions.

Photosynthesis-independent production of ROS at increased light intensity also occurs, and it is mediated by NADPH oxidase as demonstrated in rice leaves (60 min 750 μmol m^−2^ s^−1^, control 75 μmol m^−2^ s^−1^) [35]. It was revealed in Arabidopsis that NADPH oxidase and PhyB are involved in a key regulatory module controlling apoplastic ROS production during exposure to high light stress (10 or 50 min, 740 μmol m^−2^ s^−1^, control 50 μmol m^−2^ s^−1^) [36]. In this regulatory system, the increased O_2_^.−^ formation is derived from the transcriptional control of the enzyme in rice [35]. Interestingly, increased O_2_^.−^ levels were observed even after a 4-h exposure to high light intensity (4 h, 1200 μmol m^−2^ s^−1^, control 300 μmol m^−2^ s^−1^) in pea leaves [37]. This quick change in ROS levels may indicate their signalling role during the adaptation to the alterations in the light intensity. The various ROS may have specific functions in this response since exposure of Arabidopsis shoots to high light intensity (1000 μmol m^−2^ s^−1^) resulted in the accumulation of H_2_O_2_, but not O_2_^.−^ in roots [38]. Further evidence for this role of H_2_O_2_ was found in another experiment with Arabidopsis, in which high light treatment (1000 μmol m^−2^ s^−1^, control 100 μmol m^−2^ s^−1^) of certain leaves led to the accumulation of H_2_O_2_ in distant leaves not subjected to high light [39]. Besides the model plant Arabidopsis, the high light intensity (500 μmol m^−2^ s^−1^, 7 d) also induced ROS accumulation in crop species such as wheat [40]. Similarly to high light intensity, a lower level or absence of illumination can also lead to greater ROS formation, as demonstrated in leaves of Arabidopsis [41]. The regulation of ROS levels by changes in light intensity (45 min, 1500 μmol m^−2^ s^−1^, control 100 μmol m^−2^ s^−1^) may occur through the transcriptional control of the enzymes participating in their metabolism as described for ^1^O_2_ and O_2_^.−^ in Arabidopsis [42,43].

Induction of ROS formation by high light intensity is accompanied by the accumulation of various antioxidant compounds, as observed for the increasing level of carotenoids, phenols, tannins, and flavonoids with increasing altitudes and light intensity in shoots of four medicinal plant species grown at natural habitats [44]. Similar results were obtained for carotenoids, phenols, tannins, and flavonoids in shoots of other five species collected at various altitudes [45]. The role of non-enzymatic antioxidants in response to high light intensity was also corroborated in experiments under controlled environmental conditions since the GSH content was greater in wheat grown at elevated light intensity (2 weeks 500 μmol m^−2^ s^−1^, control 250 m^−2^ s^−1^) [46]. This change derived from the activation of the two enzymes of GSH synthesis (γ-glutamyl-cysteine synthase (γECS) and GSH synthase 2) at the transcriptional level. Light stress (0, 20, 60, and 90 s 1000 μmol m^−2^ s^−1^, control 100 μmol m^−2^ s^−1^) also led to greater GSH content in Arabidopsis [47]. The importance of AsA in the adaptation to high light intensity (4 weeks 1800 μmol m^−2^ s^−1^, control 180 μmol m^−2^ s^−1^) was demonstrated in ascorbate-deficient Arabidopsis mutants in which the greater accumulation of GSH compensated the lack of AsA [48]. Similarly to the modulation of GSH content, the enzymes of the D-mannose/L-galactose pathway (involved in AsA synthesis) were activated by high light at the transcriptional level [49]. The expression of the transcription factors affecting this pathway was also induced simultaneously. Besides AsA and GSH, several secondary metabolites play an important role in the defence against the harmful effect of high light. Among them, the high light intensity-induced increase in the number of flavonoids derived from the greater expression of the genes related to their synthesis [50]. Both LONG HYPOCOTYL5-dependent (HY5) and independent activation of flavonoid biosynthesis were observed in high light [51]. The anthocyanin and phenol content and the expression of the genes encoding enzymes of anthocyanin synthesis greatly increased in high light (15-day 200 µmol m^−2^ s^−1^, control: 100 µmol m^−2^ s^−1^) in Arabidopsis [52]. Carotenoids participate in the dissipation of excess light energy into heat from photosystem II in order to reduce ROS formation, and their synthesis is modulated at both transcriptional and post-transcriptional levels [53,54]. The interconversion of violaxanthin, antheraxanthin, and zeaxanthin (xanthophyll cycle) reduces ROS production at high light intensity [55]. Shade (15-day 250 μmol m^–2^ s^–1^, control: 1000 μmol m^−2^ s^−1^) also influences the amount of the antioxidant compounds as shown by the decrease in the amount of anthocyanins and the activity of enzymes of their synthesis in purple pak choi [56].

The enzymatic antioxidants also have an important role in the removal of excess ROS under high light. Under natural conditions, the activity of catalase and APX became greater at higher altitudes (greater light intensity) in four plant species [44]. In addition, the catalase, SOD, and peroxidase activities also increased with increasing altitudes in *Leymus secalinus* [57]. In a controlled environment, high light intensity activated APX and SOD in cashew (*Anacardium occidentale* L.) [58]. It had a similar effect on APX, GR, and SOD in beans [59]. This activation may exist at the transcriptional level as observed for APX, GR, and GST in wheat (2 weeks 500 μmol m^−2^ s^−1^, control 250 m^−2^ s^−1^) [46]. Interestingly, the lack of illumination for four days increased SOD, CAT, and APX activities in the leaves of *Pelargonium zonale* L. [60]. In addition, guaiacol peroxidase, catalase, and SOD activities transiently increased during the 5-day shade treatments in pak choi [61]. The activation of these antioxidant enzymes may be an indicator of greater ROS formation, as observed in Arabidopsis in prolonged darkness [41]. In summary, the antioxidant enzymes, together with the other components of the redox system, are very sensitive to changes in light intensity, and have an important role in the modulation of the redox environment of plant tissues by keeping ROS levels under control.

### 2.2. Regulation of the Redox System by Light Spectrum

Similarly to the intensity of light, its spectrum also influences the redox system [6]. Increased red:far-red ratio resulted in a greater amount of H_2_O_2_ and O_2_^.−^ in leaves of wild-type tomato plants, which effect could not be observed in *phyB* mutants, indicating the involvement of PhyB in this regulatory process [62]. Blue and yellow light induced the accumulation of H_2_O_2_ and O_2_^.−^, while red light reduced their level in comparison to white light in leaves of *Camptotheca acuminata* [63]. However, blue light decreased the amount of H_2_O_2_ in Chinese cabbage, and red light increased it in Arabidopsis, respectively [64,65]. This latter effect was connected to the light-responsive HY5 transcription factor, which mediates the effect of spectral changes on the amount of ROS by the involvement of the photoreceptors PHYs and CRYs [54,66]. These results show that the light spectrum may have a special influence on ROS in the various species, which may also depend on the interaction between light quality and other environmental factors.

The light-spectrum-dependent changes in the amount of ROS can influence the levels of the various antioxidant compounds to ensure the balance between ROS formation and removal. Correspondingly, the amount of GSH and its ratio to GSSG decreased in shoots of wheat seedlings grown in pink and far-red light compared to those cultivated in white light [67]. This change was the result of transcriptional regulation based on the reduced expression of GSH synthetase and GSSG reductase genes. The level of AsA and flavonoids increased in blue light and decreased in far-red light in lettuce [68]. The blue light-induced increase in the size of the total ascorbate pool derived from its greater regeneration in leaves of lettuce, since despite the greater expression of genes related to both its synthesis and regeneration, a corresponding change at the activity level was only observed for the enzymes of AsA regeneration [69]. UV-B-radiation resulted in the accumulation of tocopherol in *Arabidopsis thaliana* [70]. Although the highest amount of certain flavonoids (kaempferol, isoquercitrin, and quercetin) was detected in leaves of blue light-treated *Cyclocarya paliurus* plants, the total flavonoid content was also increased by red and green lights [71]. Light spectrum modulated flavonoid formation at the transcriptional level, as shown by the positive correlations between flavonoid levels and the expression of the genes encoding the main enzymes of their synthesis. Certain flavonols, such as kaempferol, quercetin, and myricetin, participate in the defence against UV radiation [72]. The biosynthesis of carotenoids is also influenced by the spectral composition of light in plants [73]. Blue and far-red light affected the amount of beta-carotene in lettuce [74]. These observations show the importance of spectral changes in the adjustment of the level of antioxidant compounds.

Besides the non-enzymatic antioxidants, the antioxidant enzymes also have an important role in the mediation of the effect of the light spectrum on the redox conditions of the tissues. The activity of the antioxidant enzymes was greater in blue light compared to the red one in common buckwheat sprouts [75]. Such a difference was also observed for SOD and peroxidase activities in ramie [76]. Increased red:far-red ratio resulted in greater SOD and lower catalase activity in the leaves of tomato [62]. Both red and blue lights increased the activity of catalase, SOD, APX, and glutathione peroxidase in *Scrophularia kakudensis* [77]. Transcriptional repression of APX and GST by blue light was found in wheat [67]. Supplemental far-red light activated the transcription of the genes encoding GR, GST, APX, and catalase, while blue light did not affect or inhibit them in barley [78]. These results indicate that the light spectrum controls the antioxidant enzymes at the gene expression level, but the effect of the individual spectral components may differ between plant species.

## 3. Regulatory Relationships between the Redox System and the miRNAs

### 3.1. Interactions between ROS, ROS-Processing Enzymes and miRNAs

**Figure 1:**
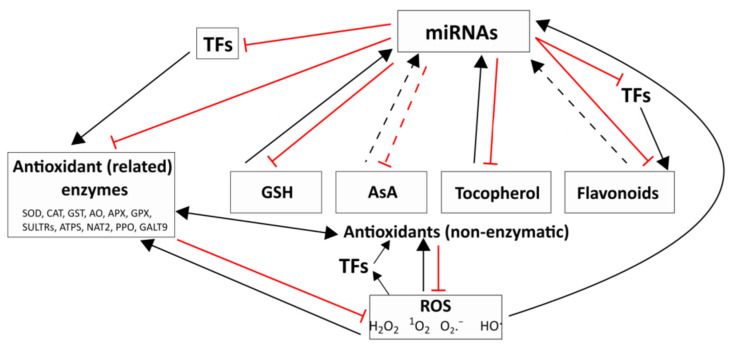
Interactions between the redox system and miRNAs. Changes in the level of reactive oxygen species (ROS) and antioxidant compounds and the activity of the antioxidant system may affect the amount of miRNAs directly or indirectly through the modulation of their biogenesis. ROS: hydrogen peroxide (H_2_O_2_), superoxide radical (O_2_^.−^), hydroxyl radical (HO^.^), singlet oxygen (^1^O_2_). Main antioxidants: glutathione (GSH), ascorbate (AsA), tocopherol, and flavonoids. Antioxidant (related) enzymes: superoxide dismutase (SOD), catalase (CAT), glutathione-S-transferase (GST), ascorbate oxidase (AO), ascorbate peroxidase (APX), glutathione peroxidase (GPX), sulfate transporters (SULTRs), ATP sulfurylase (ATPS), nucleobase ascorbate transporter 2 (NAT2), polyphenol oxidase (PPO), and galactosyl transferase 9 (GALT9). TFs—transcription factors. The solid lines indicate such relationships which are supported by previous studies cited in the text. The dashed lines show supposed relationships which should be confirmed in future experiments.

Under various stress effects, including high light intensity or shade, the expression level of numerous miRNAs was changed because of the increased ROS production [79]. The interplay between ROS and miRNAs takes place in two ways: ROS-dependent regulation of miRNA expression and control of ROS production and scavenging by miRNAs (Figure 1). In humans, the regulation of miRNA expression by ROS through miRNA biogenesis enzymes, transcription factors, and epigenetic modifications was studied extensively [80]. According to a schematic model of He and Jiang [81], ROS affect every step of human miRNA biogenesis directly or indirectly. This regulatory role of ROS is almost completely unknown in plants; however, a study suggested a relationship between ROS action and miRNA biogenesis in Arabidopsis under light stress [82]. In the review of Cimini et al. [16], 23 miRNAs were collected from various studies, which directly regulate several genes with ROS production/scavenging function in different plant species. In addition to this summary, thirteen ROS regulator miRNAs were described recently in plants (Table 1).

In cereals, miR172 regulates salt tolerance positively by targeting the *INDETERMINATE SPIKELET1* (*IDS1*) transcription factor. IDS1 can bind to the promoter of ROS-scavengers such as *APX*, *CAT,* and *GLUTATHIONE PEROXIDASE* (*GPX)* genes to repress their transcription [83]. In addition, two miRNAs, miR9674a and miR5086, were identified in wheat, which influence the salt [84] and osmotic stress tolerance [85] positively by regulating ROS homeostasis. One of the monocot-specific miRNAs, miR528, has multifaceted roles in different biological processes, such as stress response, flowering, lodging resistance, and ROS homeostasis [86]. According to Zhu et al. [87], miR528 is a hub regulator in maintaining ROS homeostasis by targeting the genes encoding copper-containing proteins, including cupredoxins, Cu/Zn SOD, PPO, laccase (LAC), and L-ascorbate oxidase (AO). In rice, the overexpression of miR528 resulted in a higher AsA and abscisic acid (ABA) content in parallel with a significant decrease in ROS and an increase in salt tolerance. These findings revealed that miR528 positively regulates salt stress tolerance via targeting *AO* gene, and, as a result, it boosts the AsA and ABA biosynthesis and ROS scavenging [88]. In dicots, several studies have been published in recent years in which the miRNA-mediated direct or indirect ROS scavenging is described under various stress effects (Table 1). In the miR775 overexpression lines, the recovery is enhanced after submergence stress, and the ROS level is reduced compared to the wild-type Arabidopsis. miR775 regulates *GALACTOSYLTRANSFERASE 9* (*GALT9*). This miR775/*GALT9* module has a prominent role in post-submergence recovery via crosstalk between the ethylene signalling and ABA biosynthesis pathways [89]. The miR164g/*MsNAC022* module can regulate the ROS scavenging system, which affects the drought stress response in apples [90]. In summary, several ROS production- and scavenging-related miRNAs and their target genes have been described in the last few years in plants; however, there are many unanswered questions about the interplay between ROS and miRNAs, mainly about the control of miRNA biogenesis by ROS.

### 3.2. Interplay between the Main Non-Enzymatic and Enzymatic Antioxidants and miRNAs

While the previous section introduced how miRNAs can regulate the level of ROS through their influence on certain ROS-processing enzymes, in this section, we would like to show the relationship between the main non-enzymatic antioxidants (GSH, AsA, tocopherol, and flavonoids), several enzymatic antioxidants and miRNAs. Both glutathione and miRNAs have multiple functions in plants, such as control of growth, development, stress responses, and redox homeostasis [95,96,97]. However, the interaction between miRNAs and GSH is not yet clarified in plants, and the possible regulation of GSH biosynthesis by miRNAs is also unknown. In human cells, there is a modulation of intracellular glutathione levels through the inhibition of its biosynthesis by miRNAs [98]. In plants, Datta et al. [99] suggest a decisive role for GSH in the miRNA-mediated regulation of defence-related genes during pathogen infection-induced oxidative stress. This assumption is supported by our previous study, in which a glutathione-related redox control of miRNAs and their targets was revealed in wheat [100]. Although the possible direct control of the two steps of GSH formation [101] by miRNAs was not investigated, the regulation of the synthesis of its precursor, Cys, by miR395 was shown in plants. It controls low-affinity *SULFATE TRANSPORTERS* (*SULTR2;1*) and *ATP SULFURYLASE* (*ATPS*) in assimilatory sulfate reduction [102,103,104]. Based on these results, a miR395-mediated indirect control of glutathione synthesis can be assumed via regulating the sulfate uptake and assimilation. Besides being an essential metabolic precursor of many biomolecules, cysteine can act as an electron donor (reduced form, cysteine) or electron acceptor (oxidised form, cystine), regulating the redox status in plant cells [105]. While there is no evidence for miRNA regulation of the ascorbate biosynthesis pathway, miR911 and miR2911 play an important role in the regulation of tocopherol biosynthesis in sunflower plants (*Helianthus annus* L.) according to Barozai et al. [106] and Hossain et al. [107]. Additionally, the positive regulatory effect of the lipid-soluble tocopherol (vitamin E) on miRNA biogenesis has been demonstrated in plants [108].

Flavonoids can act as antioxidants by scavenging ROS, but they can also chelate metals, catalysing ROS formation, which suggests a signalling modulator function [109]. Flavonoid biosynthesis involves a complex process with multiple enzymatic reactions resulting in the production of various flavonoid compounds [110]. Key enzymes and transcription factors related to flavonoid biosynthesis are negatively regulated by miRNAs [111]. Yang et al. [112] collected 25 miRNAs in countless plant species targeting structural genes involved in regulating plant flavonoid biosynthesis. In economically important soybean, miR4993 regulates the expression of *G/HFB1*, which plays a role in CHS (chalcone synthase) phosphorylation. In the same study, miR4993 was described as a regulator of UDP-glucose, flavonoid 3-O-glucosyltransferase in flavonoid biosynthesis. miR869 can influence isoflavonoid biosynthesis by targeting soybean chalcone isomerase 3 (*CHI3*) [113]. Another example is the versatility of miRNAs demonstrated by the OSmiR396-OsGRF8-OsF3H module, which regulates BHP (brown planthopper) resistance by targeting the *OsGRF8* gene in rice [114]. OsGRF8 can regulate the expression of *OsF3H* in the flavonoid biosynthesis pathway, mediating BHP resistance through a mechanism in which OsF3H positively regulates both flavonoid content and BPH resistance. In tomatoes, miR167a targets *CHI* and increases flavonoid content in the pulp and skin [115]. Six miRNAs were identified in tea (*Camellia sinensis*) regulating catechin biosynthesis [116]. Furthermore, miRNAs can regulate flavonoid biosynthesis by targeting transcription factors (TFs) [112]. The plant-specific TFs, *SQUAMOSA PROMOTER BINDING PROTEIN-LIKE* (*SPL*) genes targeted by miR156 have crucial roles in regulating various aspects of plant growth and development, including flower development, phase transition, and stress responses [117]. The miR156-*SPL9* module in Arabidopsis negatively regulates anthocyanin biosynthesis [118], which has also been observed in several other plant species [113,119,120,121,122]. In *Arachis hypogaea* and *Gossypium hirsutum*, SPLs also regulate flavonoid biosynthesis [123,124]. In light of the evidence presented, it is clear that miRNAs play a significant role in regulating the biosynthesis of main antioxidants, making them a crucial factor in the antioxidant potential of plants.

Several studies described miRNAs, which can regulate glutathione- and ascorbate-related genes such as those encoding GSTs [125,126]. The GST superfamily has a dominant role in normal cellular metabolism and numerous stress responses via the conjugation of reduced glutathione [127]. Under phosphate deficiency, the miR169j/k and the novel-miR159 negatively regulate the expression of *GST* in *Medicago sativa* roots [125]. Arabidopsis miR408 is a key player in abiotic stress response through controlling genes related to the antioxidant system, including *Cu/Zn SODs* (*CSD1* and *CSD2*) and *GST-U25* [126]. The legume-specific miR4415 targets the *AO* gene (AsA oxidation) controlling the redox state of apoplast and the cold acclimation in *Ammopiptanthus nanus* [56]. Wang et al. [128] suggested a miR156—*NUCLEOBASE ASCORBATE TRANSPORTER 2* (*HvNAT2*) module, which is involved in cadmium tolerance via enhancing the antioxidant capacity in barley. In apples, an interaction between miR171i and *SCARECROW-LIKE PROTEINS26.1* gene was described, which enhances drought stress tolerance by regulating the AsA metabolism [129]. These results indicate the crosstalk between the redox system and miRNAs in the control of various physiological and biochemical processes.

## 4. Control of miRNAs by Light

### 4.1. Regulatory Relationships between the Light Intensity and the miRNAs

**Figure 2:**
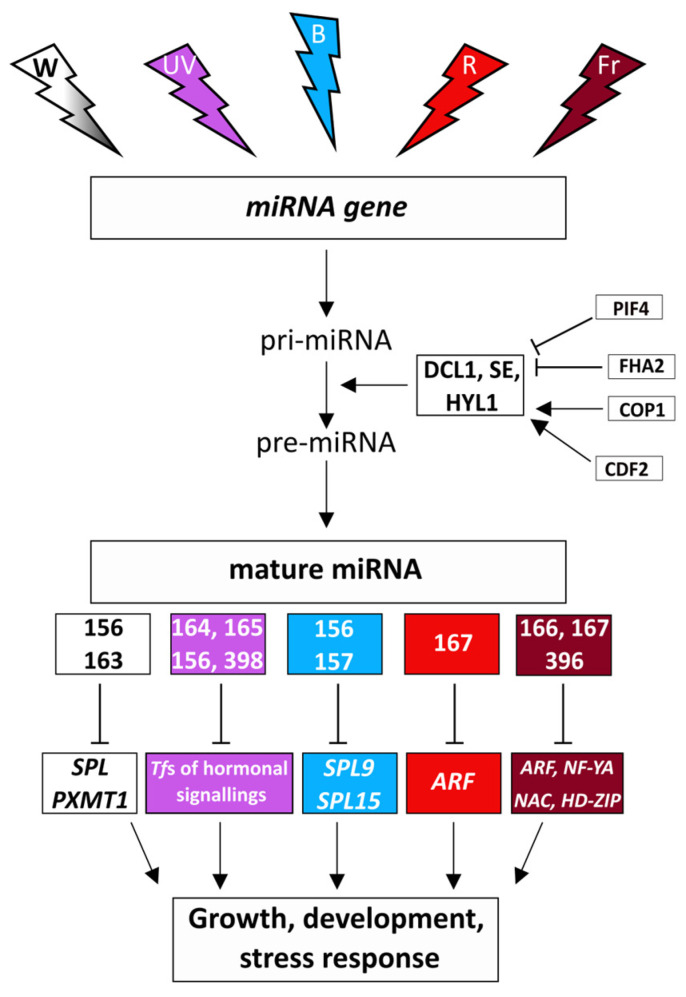
Regulation of miRNA biogenesis, miRNAs, and their target genes by the intensity of white light and its spectral composition (white light: W-white color, UV-B light: UV-purple color, blue light: B-blue color, red light: R-red color, far-red light: Fr-burgundy color). Target genes: *SQUAMOSA-PROMOTER BINDING PROTEIN-LIKE* (*SPL*), *PXMT1*: *PARAXANTHINE METHYLTRANSFERASE1*, *Tfs*: transcription factors, *AUXIN RESPONSE FACTOR* (*ARF*), *NUCLEAR TRANSCRIPTION FACTOR-YA* (*NF-YA*), *NAC TRANSCRIPTION FACTOR* (*NAC*), *HOMEODOMAIN-LEUCINE ZIPPER* (*HD-ZIP*). DICER-LIKE1 (DLC1), SERRATE (SE), HYPONASTIC LEAVES 1 (HYL1), CONSTITUTIVE PHOTOMORPHOGENIC 1 (COP1), CYCLING DOF TRANSCRIPTION FACTOR (CDF2), PHYTOCHROME INTERACTING FACTORS (PIF4), FORKHEAD-ASSOCIATED DOMAIN 2 (FHA2).

Light intensity may regulate miRNAs through the control of their transcription, processing, and function (modulation of the RNA-induced silencing complex) in plants (Figure 2) [130,131]. Thus, transferring dark-grown Arabidopsis seedlings into light resulted in the accumulation of both pri-miRNAs and the DCL1, SE, and HYL1 core processing components to high levels [132]. However, the miRNA levels did not change because of the reduction of the pri-miRNA processing activity. This latter alteration is derived from the regulatory effect of the FORKHEAD-ASSOCIATED DOMAIN 2 (FHA2) protein, which is a light-stabilized suppressor of miRNA formation in Arabidopsis [133]. FHA2 controls miRNA levels by modulating the binding of the core processing components to miRNAs. Its deficiency resulted in a greater number of matured miRNAs and a simultaneous decrease in the number of pri-miRNAs and target mRNAs. A positive regulator of miRNA biogenesis is CONSTITUTIVE PHOTOMORPHOGENIC 1 (COP1, a RING-finger E3 ligase) protein under the light in the cytoplasm, which protects HYL1 against proteolysis [134].

Interestingly, the exposure of Arabidopsis leaves to high light intensity induced systemic changes in the microtranscriptome profile of Arabidopsis roots, which indicates the transmission of stress signals (probably, certain miRNAs or unknown transmitters) to the below-ground organs [82]. HY5 transcription factor (mediating photoreceptor responses) and certain miRNAs were suggested as signal transmitters. The targets of the 17 upregulated and five down-regulated miRNAs, among others, control translation, auxin signalling, and plastid division. The number of high light-responsive miRNAs depends on the duration of treatment, as shown in Arabidopsis leaves [135]. Thus, after 3 h, 24 miRNAs (8 up and 16 down), after 6 h, 56 miRNAs (26 up and 30 down), and after 2 days, 26 miRNAs (14 up and 12 down) were affected by high light, and their major targets regulate RNA biogenesis, signalling and transcription. In another study, 2-h treatment with high light intensity increased and decreased the expression of 7 and 14 miRNAs in Arabidopsis leaves, respectively [82]. Among them, the increased expression of miR163 and miR840 was also confirmed by qRT-PCR. Based on the many-fold and small increase in the level of pri-miR163 and pri-miR840, respectively, their response to high light was controlled at different steps of their biogenesis. In *hyl1* mutant having disturbed pre-miRNA processing, the accumulation of pri-miR163 was only observed [82]. In contrast to miR163, the expression of its target, a *PARAXANTHINE METHYLTRANSFERASE* (*PXMT1*), modulates root architecture and primary root elongation) was down-regulated by high light in Arabidopsis [136]. Its repression by light could not be observed in a miR163 null mutant, but it was restored in transgenic lines expressing pri-miR163, which corroborates the regulation of *PXMT1* by miR163 (Figure 2). The effect of light on miR163 and *PXMT1* is mediated by the HY5 (a central positive regulator of photomorphogenesis) transcription factor, which accumulated in the light-exposed leaves and moved to distinct, systematic leaves in tomato [137]. HY5 regulates miR163 by direct binding to the two G/C-hybrid elements in its promoter in Arabidopsis [138]. The primary root elongation of *hy5* mutant Arabidopsis seedlings was restored and inhibited by the overexpression of miR163 and *PXMT1* genes, respectively. Besides miR163, direct binding of HY5 to miR156d, miR172b, miR402, miR408, miR775, miR858, miR869, and miR1888 promoters was demonstrated in Arabidopsis, and the expression of the 21 target genes of these miRNAs was upregulated in *hy5* knock-out mutant [139].

Similarly to high light, low light intensity also modifies the expression of miRNAs, as observed in rice [140]. Shade during the reproductive stage increased the expression of miRNAs related to the cell wall, membrane, cytoskeleton, and cellulose synthesis, and it decreased the transcription of those involved in the control of photosynthesis, carbon and sugar metabolism, energy metabolism, and amino acid and protein metabolism. The identified 16 miRNAs and their 21 targets may contribute to the improved shade tolerance and reduced yield loss. In another study with rice, the transcription of the earlier described osa-miR166c-3p, osamiR2102-3p and osa-miR530-3p, and the newly identified osa-novmiR1, osa-novmiR2, osa-novmiR3, osa-novmiR4 and osa-novmiR5 was differently affected (up- and down-regulated) by low light in two rice genotypes having different shade tolerance [141]. Consequently, the specific modulation of the target genes maintaining photosynthetic and metabolic pathways made the successful adaptation possible only to low light stress in the tolerant rice genotype but not in the sensitive one [141]. The effect of low light intensity on miRNAs was also reported in Arabidopsis, in which it decreased the level of miR156 through its inactivation by the phytochrome interacting factors and increased the expression of the *SPL* gene involved in the control of growth and development and associated with various morphological alterations characteristic for shade avoidance response (Figure 2) [142]. The effect of dark on miRNA profile was shown in *Dendrocalamus latiflorus*, in which it increased the level of miRC1, miRC22, miRC25, miRC27- 5p, and miRC27-3p, and decreased that of miRC5, miRC17, and miRC29 [143]. According to the above studies, both high and low light intensity greatly affect the expression of miRNAs and their targets, which control exists at different levels of miRNA biogenesis.

### 4.2. Spectral Control of miRNAs

Besides the intensity of light, its spectral composition also greatly affects the biogenesis and level of miRNAs (Figure 2). Thus, far-red light (1 h) resulted in tissue-specific changes in miRNA profile in etiolated soybean seedlings, as shown by the comparison of the miRNA transcriptome in cotyledon, hypocotyl, and the convex and concave sides of the apical hook [144]. Far-red light affected the expression of miR166, miR167, and miR396, involved in the control of growth, development, and auxin signalling. The involvement of miRNAs in the far-red light-dependent control of development was confirmed in the *ago1* Arabidopsis mutant, which had modified photomorphogenesis [144]. The effect of red and far-red light on miRNAs can be studied in phytochrome mutants as performed in rice since far-red light-regulated miRNAs were successfully determined by the comparison of the expression profile in wild-type and *phyB* mutant plants with disturbed light signalling [145]. Between the two genotypes, 135 miRNAs were differentially transcribed, and the role of 32 of them in the slicing of 70 target mRNAs (mainly that of transcription factors: *ARF*, *NF-YA*, *NAC*, *HD-ZIP*) was proved.

Blue light-responsive miRNAs [146] were determined by the comparison of the transcriptome in wild-type and cryptochrome2-overexpressing tomato plants [147]. Their targets participated in the control of transcription and stress response. Blue light treatment of *Brassica rapa* seedlings for one day greatly modified the microtranscriptome pattern [148]. Among others, it decreased miR156 and miR157 with simultaneous upregulation of their target transcription factors *SPL9* and *SPL15* (Figure 2). During a 4-week growth of Arabidopsis in blue light, just like the expression of a hormone signalling-related miRNA, the miR167 was modified compared to white light [149]. miR167 controls auxin response factor genes. In addition, the transition of tomato seedlings from red to blue light for 2 min led to the differential expression of 15 known and 5 predicted novel miRNAs compared to the control plants further cultivated in red light [150]. Their target genes control zeatin biosynthesis (sly-miR9472-3p–*ADENYLATE ISOAMYLTRANSFERASE*) and hormone signal transduction (sly-miR169b–*TIFY protein* and sly-miR9474–*PROTEIN PHOSPHATASE 2C*). Since the exposure to blue light lasted only 2 min, the results indicate a very rapid spectral modulation of gene expression.

A possible relationship between the red/far-red and blue light-related control of miRNA expression was indicated by the study of the effect of blue light on the miRNA profile in *phyb* mutant Arabidopsis [149]. The transcription of most investigated miRNAs (among them the ontogenetic- and morphological development-related miR160, miR165, miR163, miR402, miR168, miR172, miR170, miR166, miR167, and miR156) was greater in *phyb* mutants in blue light compared to white and red light, which led to the normalisation of phenotype and photosynthesis. The expression of *HYL1* and *DCL1* genes was also greater in *phyb*, which indicates the induction of miRNA biogenesis and function by blue light [149]. The authors assumed that this effect was not due to the induction of the blue light photoreceptor system but rather it derived from the complete inactivation of the PHYs leading to decreased negative regulation of miRNA formation by the phytochrome interacting factors (PIFs). Among them, PIF4 binds directly to the promoters of some miRNA genes and regulates their transcriptions in Arabidopsis [145]. At the post-transcriptional level, PIF4 interacts with DCL1 and HYL1 to promote their destabilization during dark-to-red light transition and controls the processing of pri-miRNAs including the photomorphogenesis-related miR160b, miR167b, miR319b, and miR848. miR319 regulates the expression of the light-signalling-related HY5, which in turn activates HEN1 (a methyltransferase stabilizing mature miRNAs), increasing the formation of mature miR319 [151]. *HEN1* expression was also affected by the photoreceptors’ PHYs and CRYs, the effect of which was probably mediated by HY5. As well as with PIF4, a blue light signalling-related cycling DOF transcription factor (CDF2) can regulate the amount of miRNAs both at the transcriptional and post-transcriptional level in Arabidopsis [152]. It can also interact with DCL1 or bind to the promoters of miRNA genes. Among the targets of CDF2 are miR156 and miR172 regulating flowering. The above results indicate the regulatory interactions between miRNAs, PIFs, and HY5.

The effect of UV-radiation (at ground level 95% UV-A and 5% UV-B) was studied in Arabidopsis grown at various altitudes (UV-radiation grows with increasing altitude) in Indian Himalayas, and 30 differentially expressed miRNAs were identified [146]. The transcription of miR169, miR8183 (16-fold), miR840, miR342, miR395, miR823, miR5653, miR781, and miR847 was greater at the higher altitude population compared to the lower one. Their target genes were associated with developmental processes, abiotic stresses (including UV), and secondary metabolites. Under a controlled environment, UV-B (8h) increased the expression of miR164, miR165, miR166, and miR398 and decreased that of miR156, miR171, miR172, miR396, and miR529 in maize seedlings [153]. Their target mRNAs encode proteins involved in the control of development, growth, and stress response. In UV-B-treated poplar plantlet, 13 up-regulated, and 11 down-regulated miRNAs were determined, which control genes encoding transcription factors and proteins involved in hormonal signaling (Figure 2) [154]. In Arabidopsis, the expression of 21 miRNAs was increased by UV-B, and their target genes controlled stress response and transcription [155]. The members of miR164, miR165, miR156, and miR398 family were affected by UV-B radiation in all three plant species, which indicate their general involvement in response to UV-B.

## 5. Light-Dependent Interaction between the Redox System and miRNAs

**Figure 3:**
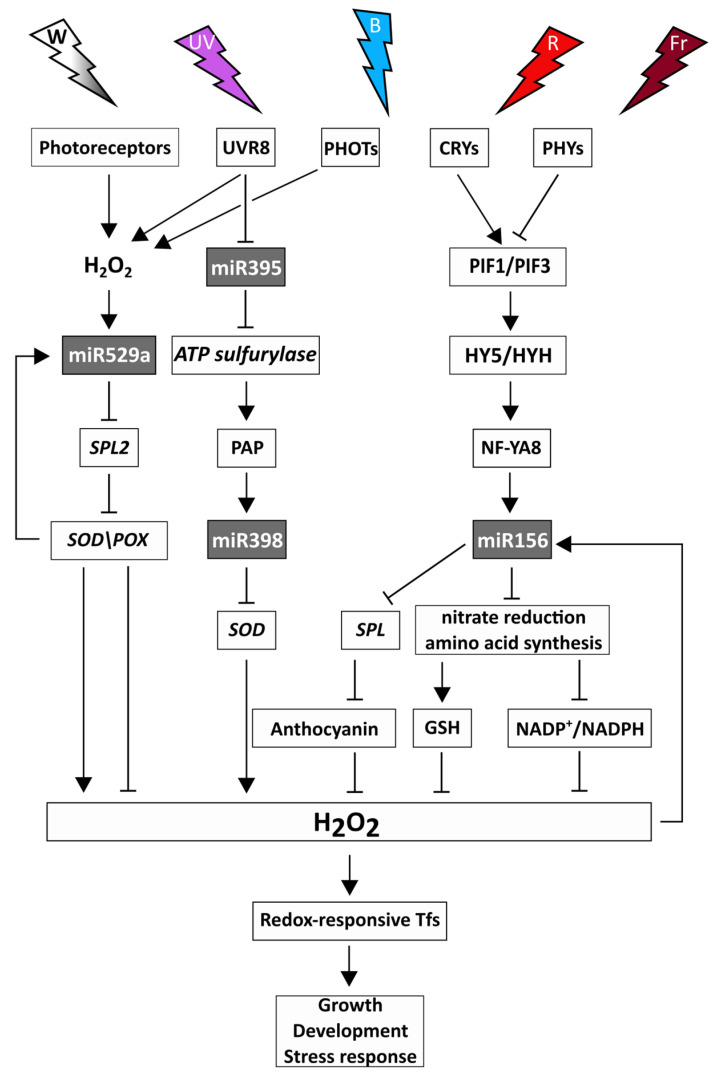
Light-dependent regulatory interactions between the redox system and miRNAs. The changes in light intensity and spectral compositions modulate the redox- and miRNA-responsive biochemical processes in plants which affect growth, development, and stress response. White light: W-white color, UV-B light: UV-purple color, blue light: B-blue color, red light: R-red color, far-red light: Fr-burgundy color. Photoreceptors: cryptochromes (CRYs), phytochromes (PHYs), phototropins (PHOTs), UV RESISTANCE LOCUS 8 (UVR8). PIF1, 3: PHYTOCHROME INTERACTING FACTORS 1, 3, HY5: LONG HYPOCOTYL5 TRANSCRIPTION FACTOR, SPL: SQUAMOSA-PROMOTER BINDING PROTEIN-LIKE, PAP: 3′-phosphoadenosine 5′-phosphate, SOD: SUPEROXIDE-DISMUTASE, POX: GUAIACOL-PEROXIDASE, HY5: ELONGATED HYPOCOTYL5, HYH: HY5 HOMOLOG, NF-YA8: NUCLEAR FACTOR Y A8, GSH: glutathione.

Since light intensity and spectrum greatly affect both the redox- and the miRNA-dependent control of the biochemical and physiological processes in plants, they may also regulate the interactions between the two regulatory systems (Figure 3). PIFs affected the expression of redox-related genes [152] and several miRNAs in Arabidopsis [149]. PIF1/PIF3 and HY5/HYH physically interact and control the transcription of ROS-responsive genes by binding to their promoters [152]. In addition, PIFs and HY5 directly bind to the promoter of several miRNA genes and regulate their transcriptions in Arabidopsis [139,145]. PIFs and HY5 may target a common promoter cis-element (G-box), as observed in Arabidopsis, and they can ensure the dynamic activation and suppression of light-responsive genes [156]. Based on these observations, PIFs and HY5 may form a light-dependent regulatory module, which coordinates the adjustment of the redox system and the miRNAs to the changes in the light intensity and spectrum. PIFs and HY5 may ensure crosstalk between the red/far-red- and blue light-dependent signalling since both PHYs and CRYs can regulate PIFs through direct binding. Both can induce the accumulation of HY5 and LONG HYPOCOTYL IN FAR-RED1 (HFR1, HY5 HOMOLOGUE) through the light-dependent inactivation of the CONSTITUTIVE PHOTOMORPHOGENIC1/SUPPRESSOR OF PHYA-105 E3 ligase complex [4]. Besides HY5, several other redox-responsive transcription factors may participate in light-responsive regulatory modules, and miRNAs targeting redox-associated genes may control their expression [16,78,137]. The assumed regulatory network of the PIF-HY5 module, redox system, and miRNAs can affect various biochemical pathways, affecting the growth, development, and stress response [6].

The role of the redox system in the mediation of the effect of high light on miRNA biogenesis was shown in Arabidopsis rosettes [82]. Using photosynthetic electron transport chain inhibitors, it was suggested that the greater pri-miRNA synthesis is associated with signals upstream of plastoquinone. However, the involvement of ^1^O_2_ produced at photosystem II could not be confirmed in a conditional fluorescent mutant (producing ^1^O_2_). Interestingly, another ^1^O_2_ signalling pathway related to β-carotene oxidation was found to participate in the regulation of miRNAs in high light. Since high light intensity decreased the expression of miR395, which is associated with the synthesis of the GSH precursor cysteine, the regulation of the redox system by miRNAs was also shown in Arabidopsis [82]. In addition, the control of miR395 by redox signalling was demonstrated in Arabidopsis mutants defective in this process, which indicates an interaction between the miR395 and the redox system [102,141].

Among ROS, H_2_O_2_ is the most stable, and has an important role in redox signalling. The expression of the H_2_O_2_-responsive miR156 [100] was affected by low light intensity, red, far-red, and blue light [142,148,149]. Overexpressing of sugarcane miR156 in Arabidopsis up-regulated key genes of nitrate reduction and amino acid synthesis (*GLUTAMINE SYNTHASE*) [157], for which processes the modulating effect of light intensity, blue, red, and far-red spectral components was shown in wheat [67]. Certain amino acids, in turn, affect the redox homeostasis through their involvement in the formation of GSH (Cys, Glu, Gly) or the consumption/production of reducing power during their metabolism (for instance Pro–NADPH); therefore, feedback control may function for the light-dependent modulation of nitrogen metabolism by miR156 and the redox system. In addition, the activation of the miR156 gene by NUCLEAR FACTOR Y A8 (NF-YA8) binding to its promoter inhibited the transition from the juvenile to the adult phase through miR156-targeted SPL proteins in Arabidopsis [158]. The miR156-*SPL9* module controls the synthesis of anthocyanins having antioxidant functions; therefore, it links the miRNA- and redox-dependent regulatory mechanisms [118]. Interestingly, *NF-YA8* was repressed by miR169q, and the overexpression of *NF-YA8* increased salt tolerance by activating the transcription of the gene encoding peroxidase1 in maize [159]. Since miR169q can be repressed by H_2_O_2_, which is degraded by peroxidases, there is a feedback modulation in this regulatory process. It is influenced by the light intensity and spectrum through the redox system-related modulation of the H_2_O_2_ levels. In addition, the NF-YA8-related regulatory process may be associated with light conditions by direct binding of HY5 to NF-Y proteins [160].

H_2_O_2_ may also be involved in the mediation of the effect of UV radiation. The participation of phototropins in this regulatory process was demonstrated in Arabidopsis since *phot1* and *phot2* single and double mutants showed a decreased accumulation of H_2_O_2_ after UV-C-induced oxidative stress [161]. In addition, UV-B increased H_2_O_2_ levels in Arabidopsis [162] and modulated the expression of several miRNAs, including miR529 in maize [153]. miR529a could be induced by H_2_O_2_, and its overexpression increased the tolerance to high H_2_O_2_ concentrations in rice, as shown by the improved seed germination rate, root tip cell viability, and chlorophyll retention. miR529 controlled the H_2_O_2_ levels by the repression of its target genes, *SPL2* and *SPL14*, leading to the increased activities of SOD and POX [163]. Since these enzymes are involved in the control of H_2_O_2_ levels, a feedback regulation can modulate the transcription of the miR529 gene under UV-B stress.

Besides ROS, the various antioxidants also have an important role in the mediation of the effect of light on miRNAs. Thus, UV-B induced the accumulation of the lipid-soluble, tyrosine-derived tocopherols [70], which are involved in the protection against lipid peroxidation and redox signalling [164], and in the control of miRNA biogenesis in Arabidopsis [108]. The positive effect of tocopherols on this process is mediated by 3′-phosphoadenosine 5′-phosphate (PAP), which inhibits the nuclear exoribonucleases (XRN). Consequently, PAP protects pri-miRNAs from degradation and supports their maturation, including miR397, miR398, and miR408. Such a control mechanism may also exist in maize, as shown by the UV-B-induced increase in the amount of miR398 [153], which negatively affects the expression of the chloroplastic SOD as observed in Arabidopsis [96]. This may alter H_2_O_2_ content and modify the expression of H_2_O_2_-responsive miRNAs described in *Brachypodium* and wheat [100,165]. Interestingly, miR398 levels were not influenced in mutants defective in redox signalling [102]. A further regulatory, light-related interaction between the miRNAs and the redox system exists through the UV-B-inducible miR395 in the above process [108,146]. This miRNA suppresses *ATPS*, catalysing the first step of PAP formation, and the reduced production of PAP in miR395f-overexpressing Arabidopsis lines may decrease the level of miR397, miR398, and miR408. In addition, the light signalling-related HY5 transcription factor directly binds to the promoter of miR408 gene, which observation confirms its control by light [139].

## 6. Conclusions

Light intensity and spectrum coordinate the function of the redox system and miRNAs through the photosensors and the transcription factors interacting with them. The fine-tuning of this regulatory network is ensured by the interaction of ROS, antioxidants, and miRNAs. The redox system and miRNAs can mediate the effect of light intensity and spectrum changes on various metabolic processes, which adjust growth and development to environmental conditions.

The limitation of the research on light-dependent interactions between the redox system and miRNAs are the lack of photoreceptor mutants in many plant species, difficulties in the determination of the interacting factors in the light signaling pathways, and the related subcellular regulatory mechanisms.

An interesting part of future studies can be the determination of the role of the proposed light-, redox- and miRNA-dependent regulatory networks in the spatial (from sub-cellular to whole plant level) and temporal (daily, seasonal, annual) control of the biochemical and physiological processes. The ROS gradients, transport of ROS, and their changes occurring at subcellular, tissue, and organ levels could be very important in this process, in which the specific function of the individual ROS species (.OH, ^1^O_2_, O_2_^.−^, H_2_O_2_) should be investigated. The possible cross-talk of ROS with reactive nitrogen and sulfur species in these networks should also be clarified. The regulatory mechanism in dicot and monocot species should be compared since specific differences may exist. The participation of plant hormones in the proposed network of ROS, antioxidants, and miRNAs can be supposed and could be clarified. Changes in the environment can modulate their regulatory interactions, which should also be studied. This interaction may have a special role in controlling the growth and development of shoot and root tips. In addition, the light-regulated networks of redox compounds and miRNAs may also influence the light signal sensing and transmitting system, but this hypothesis needs further studies in plants.

## Figures and Tables

**Table 1:** List of ROS homeostasis-related miRNAs described since 2020.

miRNA	Species	Targeted Genes	Related Stress	References
miR156	*Malus domestica*	*SPL*	Salinity	[91]
miR6024	*Solanum lycopersicum*	*NLR*	Biotic stress	[92]
miR775	*Arabidopsis thaliana*	*GALT9*	Hypoxia	[89]
miR164g	*Malus domestica*	*MsNAC022*	Drought	[90]
miR9674a	*Triticum aesivum*	*Mta/sah nucleosidase*	Drought, Salt, Osmotic	[84]
		*Serine/threonie protein*		
		*TRAP dicarboxylate transporter*		
		*AUX/IAA1*		
		*peptidase S16*		
		*RUBP activase A*		
		*TRAP dicarboxylate transporter*		
miR5086	*Triticum aesivum*	*TaTIF, TaTP, TaRPS*	Drought	[85]
		*TaRPT, TaSF, TaAP*		
miR172	*Triticum aesivum*	*IDS1*	Salinity	[83]
miR528	*Oryza sativa*	*AO*	Salinity	[88]
miR1432	*Oryza sativa*	*OsEFH1*	Biotic stress	[93]
miR408	*Glycine max*	*PR1, OXI1*	Biotic stress	[94]

Abbreviations: SPL (SQUAMOSA-PROMOTER BINDING PROTEIN-LIKE), NLR (NUCLEOTIDE-BINDING LEUCINE-RICH REPEAT PROTEIN), GALT9 (GALACTOSYL TRANSFERASE 9), MsNAC022 (NAC transcription factor), TaTIF (TRANSLATION INITIATION FACTOR SUBUNIT C), TaTP (TRANSPORT PROTEIN SECTION 16), TaRPS (RNA POLYMERASE SUBUNIT BETA), TaRPT (RNA POLYMERASE II TRANSCRIPTION SUBUNIT 13), TaSF (SUCROSE 1-FRUCTORSYLTRANSFEREASE), TaAP (AP-1 COMPLEX SUBUNIT GAMMA), IDS (INDETERMINATE SPIKELET1), AO (ASCORBATE-OXIDASE), OsEFH1 (EF-HAND FAMILY PROTEIN 1), PR1 (PATHOGENESIS-RELATED PROTEIN 1), OXI1 (SERINE/THREONINE KINASE).

## Data Availability

Not applicable.
